# Assessing training needs in health research ethics: a case study from the University of Zambia School of Medicine

**DOI:** 10.1080/11287462.2020.1853001

**Published:** 2020-12-02

**Authors:** Gershom Chongwe, Bornwell Sikateyo, Linda Kampata, Joseph Ali, Kristina Hallez, Adnan A. Hyder, Nancy Kass, Charles Michelo

**Affiliations:** a Department of Epidemiology and Biostatistics, University of Zambia School of Public Health, Lusaka, Zambia; b Department of Health Policy and Management, University of Zambia School of Public Health, Lusaka, Zambia; c Berman Institute of Bioethics, Johns Hopkins University, Baltimore, MD, USA; d Department of International Health, Johns Hopkins Bloomberg School of Public Health, Baltimore, MD, USA; e Milken Institute School of Public Health, George Washington University, Washington, DC, USA

**Keywords:** Research ethics, responsible conduct of research, bioethics, professionalism, training

## Abstract

In many settings, and perhaps especially in low-middle income countries, training institutions do not adequately prepare their students for the ethical challenges that confront them in professional life. We conducted a survey to assess the training needs in research ethics among the faculty at the University of Zambia, School of Medicine (UNZASoM) using a structured questionnaire distributed to faculty members in January 2015. The study was approved by the University of Zambia Biomedical Research Ethics Committee. Seventy-five faculty members of various ranks completed the questionnaire. It was found that 31% of the faculty had not received any research ethics training. Of those who had received training, most of them had received it through short workshops of five days or less (57.4%, *n* = 31), while only 27.7% received ethics training as a component of an academic degree and 22.2% obtained it through electronic web-based courses. While most faculty (70.7%) reported being well-prepared to guide their students in developing a research methods section of a research protocol, only 25.3% felt they were well-prepared to guide on ethical considerations. This study has demonstrated gaps in research ethics training among faculty members at UNZASoM. Mandatory instruction in research ethics among faculty and students is recommended.

## Introduction

Medical practitioners, researchers and biomedical scientists often feel ill-prepared to face many of the ethical challenges that they face in the workplace, especially in the early stages of their careers (Breslin et al., 2005; Ulrich et al., 2010). Although some tough moral issues become the subject of formal and informal debate in the public domain, there is little guidance on how best to prepare people for navigating difficult ethical challenges. In particular, researchers in health-related fields often lack a grounding in the tenets of research ethics, something that might have been inculcated at an early stage in their training. Ethics training is an essential, yet challenging element of the education of health care professionals, but ethical decision making is often difficult, situations dynamic and ambiguous, and consequences often uncertain (Mathieson, 2007). A strong grounding in the principles of research ethics and their application is therefore vital to protect patients and research participants.

The practice of science has been impacted by historical behavior, good and bad, of scientists. Instances of past abuses in particular of research participants – in both biomedical and social sciences (Richards & Schwartz, 2002; Tong et al., 2018) – have led to international guidelines with regard to ethics and human research; and international bodies also have asserted that training in research ethics is essential for all health care professionals who will be involved in the conduct of research with human participants (All European Academies, 2017; National Institutes of Health, 2011). The main goal of teaching research ethics to current and future scientists is to build capacity and strengthen local expertise in dealing with emerging ethical issues when conducting research. Despite being in existence for 50 years, the University of Zambia School of Medicine (UNZASoM) has not yet formalized the teaching of research ethics and bioethics across all disciplines and departments. A needs assessment was conducted at UNZASoM in order to begin to identify the types of teaching or training that might be most helpful. The purpose of the assessment was to determine the training needs and priorities in bioethics among members of faculty in the University of Zambia, School of Medicine.

## Methods

### Study design

A cross-sectional survey was used to determine training needs at UNZASoM by examining the level of current training, its nature and preferences for future training. This survey was conducted at the outset of an institutional bioethics partnership between UNZASoM and the Johns Hopkins Fogarty African Bioethics Training Program (FABTP) to help inform training-related components of the collaboration.

### Study setting and data collection

An original structured 25-item questionnaire with closed-ended questions was distributed by hand to 90 faculty members based at both the Ridgeway campus of UNZA and within the University Teaching Hospital in Lusaka, the largest city and capital of Zambia. The questionnaire, which was pre-tested on a small number of participants before the survey, contained closed ended (yes/no) questions, multiple choice (to choose all that applied from a list of options) as well as questions on a five-point Likert scale. Questionnaires were distributed to faculty members for them to answer in their own time with a follow up and collection by the study team after two weeks. All academic staff at lecturer, senior lecturer and (associate) professor level were eligible to participate amounting to a total of 130 eligible participants. Of these, 90 were available and agreed to participate. As a note, the School of Medicine in January 2017 was split into four independent schools, namely Public Health, Health Sciences, Nursing Sciences, and Medicine.

### Data analysis

The data were analyzed quantitatively using Stata version 14 (StataCorp, College Station, Texas, USA). There were multiple choice questions, which were subjected to descriptive analysis and a self-rated five point Likert scale. Frequencies were calculated for all questions, while tables and figures were prepared to illustrate the findings. Cuzick's test for trend (nptrend in Stata) was used to test for trends across ordered categories. Pearson's Chi-square or the Fisher's exact Chi-square test were used for cross- tabulations, as appropriate. An investigator-led multivariable backward stepwise logistic regression with a threshold set at a *p* value of 0.20 was used to examine the association between history of receiving ethics training and other characteristics. The independent variables analyzed in the regression model were the rank of the faculty member, reported capacity to teach research methods, capacity to teach research ethics, disciplinary background, and number of peer-reviewed papers ever published. We used the Akaike (AIC) and Bayesian (BIC) information criteria for model diagnostics. A *p* < 0.05 was considered statistically significant.

### Ethical considerations

Those who returned completed questionnaires were assumed to have consented to participation, as indicated in the short written disclosure and information sheet accompanying each questionnaire. Only anonymized data were used for analysis. The study was approved by both the Johns Hopkins School of Public Health Institutional Review Board and the University of Zambia Biomedical Research Ethics Committee (UNZABREC).

## Results

Seventy-five people from 12 departments of UNZASoM responded to the survey (Table 1). Most respondents were from the Department of Public Health (22.7%), followed by Nursing Sciences (21.3%). Out of this, 46 (61.3%) were junior lecturers (Grades III-I), 21 (28.0%) were senior lecturers, 8 (10.7%) were professors. Faculty from two Departments did not respond. The total overall response rate (those who responded compared to those to whom a questionnaire was distributed) was 83.3% [75/90].

**Table 1.** Distribution of respondents by name of department and rank, University of Zambia, School of Medicine.

Department	Primary rank				Total
	Lecturer	Senior lecturer	Associate professor	Full professor	
Biomedical sciences	3	0	1	0	4
Physiological sciences	3	0	0	1	4
Public health	15	0	1	1	17
Nursing sciences	8	8	0	0	16
Physiotherapy	3	4	1	0	8
Surgery	1	2	0	1	4
Psychiatry	1	3	0	0	4
Paediatrics	1	0	0	1	2
Pathology and microbiology	2	3	0	1	6
Internal medicine	1	0	0	0	1
Anatomy	1	1	0	0	2
Pharmacy	7	0	0	0	7
Total	46	21	3	5	75

### Ethics training among faculty members

**Figure 1.**
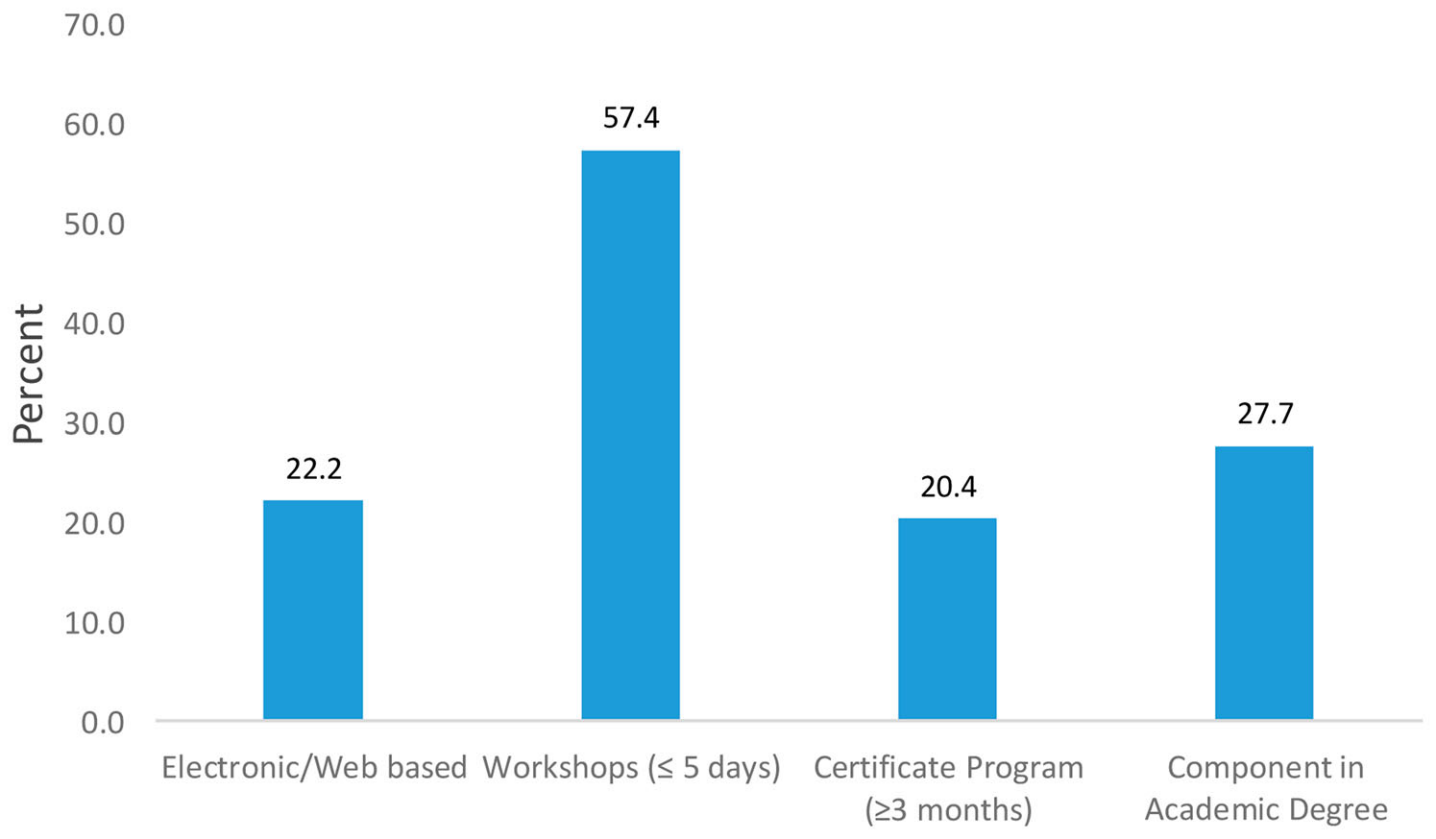
Ethics training received by Faculty Members at University of Zambia, School of Medicine (*n* = 54). Note: Some participants had received more than one type of training.

Sixty-nine percent of the respondents reported having received ethics training in some form, while 31% reported not having received any ethics training. Of those who had received some training, the majority had received the training through short workshops of five days or less (57.4%, *n* = 31), while 27.7% had received ethics training as a component of an academic degree (27.7%) and/or through electronic web-based courses (22.2%) (Figure 1). Among participants who had received training through short workshops, six (19.4%) of them also reported having had training through a web-based course, four (12.9%) had also had training through an academic degree, while two (6.4%) reported having had research ethics training lasting three months or more in addition to the short-term training. A similar proportion of respondents had received their ethics training within Zambia (44%), as who received it outside Africa (41%); 15% received their training elsewhere within Africa.

When asked to state what kind of ethics training they preferred, most of faculty members preferred the training to be in the form of formal classes or a course (61.3%), followed by periodic in-person lectures (54.7%), workshops (49.3%) and lastly electronic/web-based course (18.7%). A similar pattern emerged when respondents were asked to recommend a format for student training.

Regression modeling showed that, controlling for the rank of the faculty member and educational background, those who reported having higher numbers of publications were more likely to have received ethics training [(AOR 8.73 95%CI: 2.09, 36.4, *p* value 0.003) for those with 6–10 publications, and (AOR 20.4 95%CI 2.30, 181.3, *p* value 0.007) for those with more than 10 publications]. We found that rank and training background (whether basic science, epidemiological or social-behavioral science) were not significantly associated with having received training (Table 2).

**Table 2.** Relationship between a history of ethics training and other factors.

Title	Number	Crude odds ratio (95%CI)	*p* value	Adjusted odds ratio OR (95%CI)	*p* value
Rank					
Lecturer	46	1		1	
Senior lecturer	21	3.85 (0.99, 15.0)	0.051	–	
Professor	8	1.93 (0.35, 10.6)	0.451	–	
Number of publications					
0–5	30	1		1	
6–10	16	19.6 (2.29, 168.3)	0.007	8.73 (2.09, 36.39)	0.003
>10	27	10.5 (2.5, 42.5)	0.001	20.4 (2.30, 181.30)	0.007
Capacity to guide students in research ethics					
No	25	1		1	
Yes	50	0.23 (0.08, 0.65)	0.006	–	
Capacity to guide students in research methods					
No	22	1		1	
Yes	53	0.29 (0.10, 0.84)	0.022	0.35 (0.10, 1.27)	0.11
Basic science researcher					
No	55	1		1	
Yes	18	0.94 (0.29, 3.06)	0.915	–	
Behavioral science researcher					
No	60	1		1	
Yes	13	1.72 (0.49, 6.03)	0.398	–	
Epidemiological or clinical researcher					
No	18	1		1	
Yes	55	0.39 (0.13, 1.18)	0.096	–	

### Importance of research ethics

The vast majority of respondents (94.7%) strongly agreed that research ethics was important to research participants, and 88% disagreed that research ethics is only applicable to clinical trial research (Table 3). The majority (68%), however, either strongly disagreed (42.7%) or disagreed (25.3%) that research ethics is well understood by Zambian researchers (Table 3).

**Table 3.** Perceptions of Faculty Members at the University of Zambia, School of Medicine about the importance of research ethics.

	*n*	%
Research ethics is important to research participants		
Strongly Disagree	–	–
Disagree	–	–
Neutral	2	2.7
Agree	2	2.7
Strongly Agree	71	94.7
		
Research ethics is well understood in Zambia		
Strongly Disagree	32	42.7
Disagree	19	25.3
Neutral	17	22.7
Agree	5	6.7
Strongly Agree	2	2.7
		
Research ethics is only applicable to clinical trial research		
Strongly Disagree	61	81.3
Disagree	5	6.7
Neutral	3	4.0
Agree	2	2.7
Strongly Agree	4	5.3

### Supervision of students

Almost all respondents (97.3%) reported supervising students who carry out research.

Given that students are required to include a section on ethical considerations in the methods section of their protocols, we asked faculty members how well prepared they felt in supervising or scrutinizing these components of the proposal. While the majority of faculty (70.7%) stated that they were well prepared to guide the students in the research methods sections in the protocol, only 25.3% expressed confidence in their ability to guide the students on the ethical considerations section of the protocol (Table 4). However, there was no association between rank of lecturer and confidence in guiding students in bioethics, (test trend *p* = 0.07, not shown). A test for trend showed a positive relationship between having received any ethics training and a self-rated capacity to supervise a student in the ethical considerations section of a protocol (*p* = 0.001, not shown).

**Table 4.** Perceived preparedness of Faculty Members in supervising students in research methods and ethics of student protocols.

	Research methods		Ethical considerations	
	*n*	%	*n*	%
Prepared	53	70.7	19	25.3
Neutral	16	21.3	31	41.3
Unprepared	6	8.0	25	33.3
Total	75	100.0	75	100.0

## Discussion

This study found that, while almost all respondents (UNZASoM faculty members) felt that ethics training was important, only one-third of the faculty, almost all of whom supervise students conducting research involving humans, report not having received training in research ethics. In addition, having received no training was not associated with rank or professional background. Furthermore, among those who had received training, most had received only short-term training in research ethics, lasting up to a week, while a small number of faculty had additional training of three months or more in duration.

That so many research faculty had not received training is similar to findings from Egypt and Saudi Arabia, where up to half of the faculty in some universities were found not to have been trained in research ethics (El-Dessouky et al., 2011; Kandeel et al., 2011). Our results show that most of the training that UNZASoM researchers reported receiving was relatively short, suggesting that longer training may not have been available. However, there have been an increase in the number of funders providing short-term workshops in LMICs. While short-term training clearly is an enormous contribution compared to no training at all, it is not clear whether short-term training is enough to increase capacity in research ethics to the level needed of a research institution or of individuals who supervise students in the conduct of human subjects research. A recent rapid assessment of the broader research ethics environment at UNZASoM has reinforced the need for training and also highlighted other areas for potential systems strengthening (Hyder et al., 2017). These findings come at a time when funders of health research have become increasingly involved in promoting ethics training, (Ali et al., 2012), providing potentially more opportunities for those who have received no training to be exposed, and for those only exposed to short-term training to potentially undergo training in greater depth.

Indeed, having a primary goal of providing *any* training should be an important priority for those who conduct their own research and for those who supervise the conduct of others. Fortunately, web-based training in research ethics has been shown to offer similar results in terms of knowledge accumulation compared to on-site training in some contexts (Aggarwal et al., 2011), allowing more options in how training goals might be realized. Although formal classes or a workshop were the preferred mode of training among our participants, many examples exist where web-based portals have delivered academically rigorous coursework in research ethics that is of a high standard (Callier et al., 2017; Ellenchild Pinch & Graves, 2000). However, these systems depend on reliable internet infrastructure, which is not the case still in many LMICs, including Zambia.

The teaching and supervision of students in research ethics requires a firm grounding in the subject among members of the faculty. While a number of faculty members, mostly in the Department of Public Health (now School of Public Health), were well trained in bioethics, the same was not true for faculty from other departments. Grounding of students in research ethics from the undergraduate to the postgraduate level has a potential of entrenching research ethics culture in their practice, and will likely produce faculty that have had an opportunity for more training and hence more capability of supervising others. In the United States, the National Institutes of Health (NIH) and other agencies have mandated, for certain types of students and researchers who receive NIH support, training in responsible conduct of research (RCR) (National Institutes of Health, 2011; Plimpton, 2009). In Zambia, the National Health Research Authority is mandated to promote the training of researchers through the National Health Research Act (Parliament of Zambia, 2013). However, no official policy makes it mandatory for researchers to have ethics training before research is conducted. The need to design and put into effect formal instruction in RCR for faculty and students in all the departments at UNZASoM is evident from the lack of confidence expressed by some faculty in their ability to properly guide students in this area.

## Limitations

The results of this study cannot be generalized to other learning institutions in the country and elsewhere because of the nature of the sampling. Those outside this focus (including faculty at the Great East Road campus) were not considered for inclusion in the study. Furthermore, the dataset lacked some important demographic information that would have helped to understand the population better. However, given that UNZASoM has more international research collaborations than many other institutions in the region, it is reasonable to speculate that these findings may be conservative in representing the number of faculty members who have been trained in research ethics. This study has highlighted important challenges and opportunities that can be harnessed to understand and address institutional research ethics capacity both at UNZASoM and similar institutions.

## Conclusion

This study has highlighted important gaps in research ethics capacity in a lower-middle income country research and teaching institution that need to be addressed. There is need to develop strategies for addressing further the identified gaps in institutional research ethics training and capacity at UNZASoM and other similarly situated institutions in the country and region.

## References

[CIT0001] Aggarwal, R., Gupte, N., Kass, N., Taylor, H., Ali, J., Bhan, A., Aggarwal, A., Sisson, S. D., Kanchanaraksa, S., Mckenzie-White, J., Mcgready, J., Miotti, P., & Bollinger, R. C. (2011). A comparison of online versus on-site training in health research methodology: A randomized study. *BMC Medical Education*, *11*(1), 37. 10.1186/1472-6920-11-37 21682858 PMC3141795

[CIT0002] Ali, J., Hyder, A. A., & Kass, N. E. (2012). Research ethics capacity development in Africa: Exploring a model for individual success. *Developing World Bioethics*, *12*(2), 55–62. 10.1111/j.1471-8847.2012.00331.x 22708713 PMC3393778

[CIT0003] All European Academies (ALLEA). (2017). *The European code of conduct for research integrity*.

[CIT0004] Breslin, J. M., Macrae, S. K., Bell, J., Singer, P. A., & University of Toronto Joint Centre for Bioethics Clinical Ethics. 2005. Top 10 health care ethics challenges facing the public: Views of Toronto bioethicists. *BMC Medical Ethics*, *6*(1), E5. 10.1186/1472-6939-6-5 15978136 PMC1180442

[CIT0005] Callier, S. L., Hertelendy, A., Butler, J., Harter, T. D., Firmani, M., & Goldstein, M. M. (2017). Going online: A pedagogical assessment of bioethics distance education courses for health sciences professionals. *International Journal of Online Pedagogy and Course Design (IJOPCD)*, *7*(1), 57–70. 10.4018/IJOPCD.2017010105

[CIT0006] El-Dessouky, H. F., Abdel-Aziz, A. M., Ibrahim, C., Moni, M., Abul Fadl, R., & Silverman, H. (2011). Knowledge, awareness, and attitudes about research ethics among dental faculty in the Middle East: A pilot study. *International Journal of Dentistry*, *2011*. 10.1155/2011/694759 PMC3132601 21754933

[CIT0007] Ellenchild Pinch, W. J., & Graves, J. K. (2000). Using web-based discussion as a teaching strategy: Bioethics as an exemplar. *Journal of Advanced Nursing*, *32*(3), 704–712. 10.1046/j.1365-2648.2000.01531.x 11012815

[CIT0008] Hyder, A. A., Deutsch-Feldman, M., Ali, J., Sikateyo, B., Kass, N., & Michelo, C. (2017). Rapid assessment of institutional research ethics capacity: A case study from Zambia. *Acta Bioethica*, *23*(1), 35–46. 10.4067/S1726-569X2017000100035

[CIT0009] Kandeel, N., El-Nemer, A., Ali, N. M., Kassem, H., El-Setouhy, M., Elgharieb, M. E., Darwish, M., Awadalla, N. J., Moni, M., & Silverman, H. J. (2011). A multicenter study of the awareness and attitudes of Egyptian faculty towards research ethics: A pilot study. *Journal of Empirical Research on Human Research Ethics*, *6*(4), 99–108. 10.1525/jer.2011.6.4.99 22228064

[CIT0010] Mathieson, K. (2007). Towards a design science of ethical decision support. *Journal of Business Ethics*, *76*(3), 269–292. 10.1007/s10551-006-9281-4

[CIT0011] National Institutes of Health. (2011). *Update on the Requirement for Instruction in the Responsible Conduct of Research*. In Office for Extramural Research (Ed.), NOT-OD-10-019. Department of Health and Human Services (HHS).

[CIT0012] Parliament of Zambia. (2013). National Health Research Act 2013. *Laws of Zambia*, Act No. 2 of 2013, 27.

[CIT0013] Plimpton, S. (2009). National Science Foundation. Responsible conduct of research. *Federal Register*, *74*, 42126–42128. https://www.govinfo.gov/content/pkg/FR-2009-08-20/pdf/E9-19930.pdf

[CIT0014] Richards, H. M., & Schwartz, L. J. (2002). Ethics of qualitative research: Are there special issues for health services research? *Family Practice*, *19*(2), 135–139. 10.1093/fampra/19.2.135 11906977

[CIT0015] Tong, S. F., Tong, W. T., & Low, W. Y. (2018). Ethical issues in qualitative data collection among vulnerable populations in healthcare setting. In Cees Th. Smit Sibinga (Ed.), *Ensuring research integrity and the ethical management of data* (pp. 80–97). IGI Global. 10.4018/978-1-5225-2730-5.ch005

[CIT0016] Ulrich, C. M., Taylor, C., Soeken, K., O’Donnell, P., Farrar, A., Danis, M., & Grady, C. (2010). Everyday ethics: Ethical issues and stress in nursing practice. *Journal of Advanced Nursing*, *66*(11), 2510–2519. 10.1111/j.1365-2648.2010.05425.x 20735502 PMC3865804

